# On Temperature in Consumption, in Reply to Dr. Sutton

**Published:** 1815-11

**Authors:** I. Buxton


*For the London Medical and Physical Journal.*

### *On Temperature in Consumption, in Reply to Dr. Sutton;* by I. Buxton, M.D.

OBSERVING in your Journal for August, that Dr. Sutton has answered my reply to his observations on Warm Temperature in Consumption, I find it necessary again to trouble you on the same subject. In this answer, Dr. S. has brought several charges against me which it is requisite that I should repel in the first place. My doing this is the more needful, because, if Dr. Sutton's charges are substantiated, my statements may be considered incorrect, which I can safely maintain they are not. With respect to consumption in hot climates, I am charged (p. 92) with having preferred "inference to positive testimony," and with underrating the frequency of consumption, "although Dr. Lempriere has given given a table of deaths by the disease." Any one reading the assertions of Dr. Sutton would suppose that I had paid no attention to Lempriere, but had purposely quoted other authors and avoided him. Now the fact is, that, in the Number of the Monthly Magazine for Nov. 1814, p. 301, I have carefully cited Lempriere, and have given the numbers as mentioned by him in that very table to which Dr. S. alludes; and from this table have shewn, that not one person in twenty-five dies of consumption in Jamaica. I have, likewise, brought forward the best medical writers on the diseases of hot climates, and have noticed that none, besides Lempriere, mentions consumption. And hence, from the positive testimony of Lempriere, and from the silence of other authors, have deduced the fact, "that consumption is rare in hot climates." Is this preferring inference to positive testimony? I brought forward, in the most conspicuous manner, all the positive testimony which I could then collect among medical authors. Nor has Dr. Sutton informed me where positive testimony was to be found, which I have neglected. At any trial, all the positive and all the circumstantial evidence is produced; and, from the whole, a conclusion is drawn. I have done the same, and produced the whole evidence, and hence have drawn my conclusion. Is this preferring inference to positive testimony?—But how does Dr. Sutton prove that consumption is *not* rare in hot climates? First, by setting aside, on his own authority, and without any proof of delinquency, every writer on the diseases of hot climates besides Lempriere, and these by the following demonstration: "Lempriere (says Dr. S.) asserts, that 'among the negroes and people of colour, it (consumption) is of frequent occurrence.' This (continues Dr. S.) he states in comparison to its infrequency among the white inhabitants, but which shews the disease to prevail more than Dr. Buxton, by his inferences, is led to conclude." I suppose that Dr. S. gives the first paragraph as a quotation from a private communication made to him by Lempriere. The term frequent is relative. Sixty respirations in a minute would be frequent; sixty pulsations would not be so. Lempriere, in this passage, affirms the frequency of consumption among the negroes and people of colour, not in relation to its occurrence among white people in England, but among the white population of Jamaica. In this respect, it may be frequent; and yet may be, and actually is, rare with relation to its occurrence in Britain. This brings us to no other conclusion, than that consumption, though rare in Jamaica, when it does occur, is chiefly met with among negroes and people of colour. And this is what Lempriere has asserted more than once in the book which he published on the Diseases of Jamaica.

maica. In that publication, consumption is hardly mentioned throughout two octavo volumes, whatever may be the class of people of whom he is speaking, whilst fever of different kinds and a variety of abdominal complaints continually meet the eye. Dr. S. in this passage, twice blames me for "preferring inference to positive testimony, although Dr. Lempriere has given a table of deaths by the disease (consumption) as it occurred in Jamaica." This charge I have refuted. But it is curious, that, in the last of the passages in which I am thus accused, Dr. S. is guilty of the very fault with which he charges me. Instead of trusting to the reckoning of Lempriere's table, he gives the above quotation, (a quotation acknowledged to be not a position, but merely a relative, assertion,) and then *infers*, that the disease prevails more in Jamaica than I am led to conclude; whilst I quote the positive testimony of the table without inference, and thence shew that only one death in twenty-five is caused by consumption in that island. If Dr. S. had inserted his own name in this accusation, instead of mine, he would have been correct. Looking to Lempriere's table alone, I consider myself perfectly justified respecting hot climates; and, unless Dr. Sutton can shew, that in them more than one death in twenty-five is caused by consumption, I must be allowed to consider the disease as rare, and that I have not underrated it by following Lempriere's table.

Respecting the mild climates, I am accused of relying on Cleghorn and Domeier in their accounts of Minorca and Malta, and of asserting consumption to be "a disease of much more frequent occurrence" in England than either in Madeira or Sicily.

If the reader will take the trouble of looking at the Monthly Magazine for December, 1814, he will find that I have *not relied* on either of these authors exclusively; nay, that I have not quoted so much from them, as I have from Irvine and Gourlay, on whose testimony *alone* Dr. Sutton is inclined to rely. Besides, has Dr. S. proved Domeier or Cleghorn ignorant, partial, or unworthy of credit? He has not made the attempt: yet he would throw aside their testimony!

To advert to the latter part of the charge; my assertion in the Monthly Magazine is "that consumption is not unfrequent in mild climates." This, I explained to mean that the disease occurs oftener in the mild than in the hot countries, but not so often as in our own climate. The proposition was a conclusion deduced from comparing authors who had written respecting Spain, Minorca, Malta, Sicily, and Madeira. The first two authors had not mentioned consumption; the third said that consumption was not frequent in Malta; Irvine and Gourlay affirmed its frequency in

4 Sicily

Sicily and Madeira. I thus brought forward what evidence I could collect respecting this point; and, from comparing the whole together drew the above conclusion.

Dr. Sutton has pursued a different mode of forming his judgment. The first three writers he has entirely rejected; but without assigning any reason for his rejection. He wishes the whole of the controversy to rest on Irvine and Gourlay; and even here he selects merely those passages which speak most strongly of the frequency and fatality of consumption in those countries, whilst all that they have said of a qualifying nature he carefully keeps out of sight. So that whoever reads his paper without consulting these authors themselves, would, perhaps, draw the conclusion which he wishes them to form; viz. that consumption is as frequent in Madeira and Sicily as it is in England. He, however, sedulously guards against making this assertion, and will guard against doing so. Irvine and Gourlay have unfortunately given no lists of diseases and deaths. In consequence of this omission, Dr. Sutton, by confining himself to general expressions, and by avoiding particular assertions, might, perhaps, still keep the field in these two islands, if we had not other testimony to which to resort. In England, consumption is more fatal than any other disease. But, if any one will read the little volumes of Irvine and Gourlay, he will find, by the general tenor of these books, that consumption is not the leading disorder of either Sicily or Madeira.* Fever is evidently spoken of by Irvine as by much the most destructive disease in Sicily. Am I then wrong in believing consumption to be more frequent in England than in Sicily or Madeira? If each person who is to judge of the controversy between us would read the books themselves, I would willingly trust to Irvine and Gourlay alone, and am convinced that the conclusion I have drawn would generally be formed. But could Dr. Sutton prove (what he never will be able to prove) that consumption is as common in those islands as it is in England; still, other warm climates are to be taken into the account, such as Spain, Malta, and Minorca. But these Dr. S. studiously keeps in the back-ground. Is it by

* We cannot, on this occasion, refrain from expressing our surprise, that a paper on the climate of Madeira, inserted in our Journal, should have been overlooked; especially as we believe that paper contains the first hint of an artificial climate in Great Britain, the source of the present interesting controversy. It also explains the cause of the contradictory evidence concerning the prevalence of consumption in Madeira.—See the conclusion of a Letter from Dr. Adams, of Madeira; communicated to us by the late Dr. Willan, Vol. V. p. 307.—Edit.

 selecting selecting authors on one side of the question only, that a correct judgment is to be framed of mild climates in general.

But I happen now to have positive testimony to which I can appeal, and which will prove, that consumption is far less common in mild countries than it is in England. I have authority for stating, that in this island consumption is as frequent in the army as it is in civil life. But, when our troops were lately in Spain, Portugal, and Sicily, this disorder was of much more rare occurrence among them in each of these countries, than among the soldiery in England. It is to be observed, that Sicily, the island on which Dr. Sutton lays so great a stress is included in this enumeration. This testimony is positive and conclusive, that, although consumption may not be "unfrequent in mild climates," it is not nearly so prevalent in them as it is in England.

In the same page, I am charged with being "very anxious to impress my readers with the opinion, that this country is pre-eminent in its sufferings respecting consumption." If this passage means any thing, it means that I have overrated consumption in England. Yet, Dr. Sutton has not ventured to make this charge in direct terms. I asserted, that "consumption is very frequent in this country;" and I proved my assertion by the most authentic registers of deaths which I could procure. From these I shewed that more than one person in five died of this disorder in England. I likewise asserted, that the disease is "extremely frequent in other countries, whose temperature does not materially differ from our own."—*Monthly Magazine, Jan.* 1815, p. 521. Dr. Sutton has not attempted, nor will he attempt, in a direct manner to disprove these assertions, although he may carp at them. But, if he cannot disprove my statement, why should he throw out such an inuendo as the above?

Dr. Sutton subsequently corrects (p. 95 and 97) what he is pleased to charge on me as an error respecting the climate of Constantinople during the winter, by asserting that "the winter of Constantinople is full as severe as in England." It is to be observed, that this is an assertion merely, and is unsupported by any proof. When I have corrected the errors into which Dr. S. has fallen, I have invariably brought forward proofs, so as to demonstrate, that what I considered as errors or mistakes, actually were such. But, as Dr. Sutton has given no proof whatever, I must say, that the great triumph which he expresses in having (as he imagines) detected me in a single error is rather misplaced; since, if he consults the best authors who have written respecting Turkey, he will probably find, that his unsupported assertion is incorrect, and that "the winter of Constantinople is *not* full as severe as in England."

But

But I need not wonder that Dr. Sutton brings charges against me, when I observe that he charges seven out of eight of the authors whom I had quoted respecting hot climates with "heedlessness" and "prepossession," because they did not speak of consumption as occurring in tropical countries. I found these authors considered as worthy of credit, and accordingly employed their writings. Dr. Sutton has unfortunately forgotten to bring any proof of his charge. It certainly is not my business to vindicate them from the accusation. Perhaps, as Dr. Sutton has not attempted to prove his assertion, there was no necessity for any vindication whatever. But, as the charge had been made, I considered it proper that some one of the accused might, if he pleased, repel the accusation of Dr. S.; and accordingly wrote on this subject to Mr. Johnson, author of "The Influence of Tropical Climates on European Constitutions." The following are extracts from his reply. "The circumstance of pulmonary phthisis being hardly ever alluded to by myself or other writers on the diseases of tropical climates, might have been a sufficient evidence of the comparative infrequency of the complaint in those regions, instead of subjecting us to the charge of heedlessness and prepossession by a *fireside* traveller, who has an opposite hypothesis to support, or a controversy to maintain; but, as an ungenerous and *unjust* construction has been put on our silence, those of us who yet remain to tell the tale shall speak for ourselves and our brethren. During a period of seventeen years, I have practised and observed in every parallel of latitude from 65° north to 40° south, and in every degree of longitude from the West Indies to Canton in China. In addition to this, when preparing my work for the press, I examined with great care, at the Transport Office, the journals of all the naval medical officers who had served between the tropics during the last twenty-five or thirty years. With this experience and these materials, my evidence, as a neutral character, who has no theory to support, will probably weigh as heavy as the 'ipse dixit' of Dr. Sutton, who has no local experience of the subject, and is a professed partizan."

Mr. Johnson then proceeds to state, that the result of his observations has been, that in tropical situations diseases of the abdomen prevail much more than those of the chest, whilst, in climates like our own, diseases of the chest are most prevalent; and illustrates his observations by the following statement:—"I have served, for instance, in a ship of the line on the coast of Coromandel and in the English Channel. In the former situation, there would not be one pulmonic complaint to fifty others; and a case of phthisis pulmonalis would not be seen from the beginning to the end end of the year. In the Channel, on the contrary, pulmonic complaints would fill up two-thirds of the lists (excluding accidents and venereals) and every month there would be men invalided for phthisis pulmonalis." Surely, that must be a bad cause, where the party is desirous that seven-eighths of the witnesses should be set aside, merely because he asserts, without proof, that they are heedless and prepossessed.

Dr. Sutton observes, at the commencement of his paper, that in a former communication he "endeavoured to shew, that we had as good grounds for imitating the atmosphere of the colder climates in the cure of this disease as for attempting it by a high temperature, if we took the infrequency of the disease in different climates as the standard by which the treatment, as to temperature, should proceed." He then says, that he also "considered it far from proper to infer, because a disease is infrequent in any stated country, when it does actually occur there, that it should of course be mild or more manageable than in a country in which it occurred frequently? and thus, because "cold might contribute much to occasion a disease, that, therefore, such a disease required to be treated in a warm atmosphere as its opposite."

If Dr. Sutton had fully proved each of these points, this would not have been sufficient to shew, that warmth of temperature was otherwise than beneficial in consumption. I rested the main stress of the argument on facts and experiments; experiments made on a large scale every year by Nature herself, and occasionally imitated by medical men in warm apartments.

But I have before demonstrated, that Dr. S. has not proved even the first of these positions. That consumption is rare in hot climates is shewn directly or indirectly by every writer on the diseases of those countries. But Dr. S. has completely failed in his attempt to prove the disease rare in cold countries. In Holland, the testimony is balanced; one writer affirming the disease to be frequent, another uncommon; in the northern part of North America, it is balanced; in Russia, half the population pass their winter in a temperature much exceeding 60°, and the remainder pass half their time in the same; in Iceland, the disease is frequent; in Greenland, it is frequent. Yet, Dr. Sutton, arguing merely from *ex-parte* evidence, asserts that this "unfrequency of the disease" in cold climates is "a deduction from facts!" It is true, that the doctor makes a kind of apology for speaking thus, by saying, that "this was a deduction from facts, acknowledged to be tolerably accurate by parties who favoured the hot treatment." Thus endeavouring to pass off the *ex-parte* evidence on which he relied, by speaking of it as an *argumentum ad hominem.* A lawyer, in pleading, will, if possible, possible, gain a victory for his client, although totally at the expence of truth. With him an *argumentum ad hominem* is perfectly fair. But the philosopher, who is in search of truth, will never rest satisfied with this argument alone; particularly, if there be other evidence to which he can have recourse. Dr. Sutton, if in search of truth, should not have rested satisfied with the concessions of an opponent; but should have consulted writers on cold climates for himself, before he drew the positive conclusion, which he has given above; and then he would have found the evidence as I have just now stated it, evidence very far from sufficient to warrant the conclusion which Dr. Sutton has drawn. But even the *argumentum ad hominem* must totally fail Dr. S., because the concession on which he grounds this argument was not made by myself. If it had been made by me, it might have been employed with propriety when Dr. S. was writing against me; but no concession, by any other individual, can be employed against myself; the *argumentum ad hominem* must here, therefore, be perfectly futile.

In my former letter in your Journal, I fully answered what Dr. S. brought forward respecting cold climates; he has not attempted to answer the observations I then made; in truth, they were a plain statement of facts, and therefore unanswerable. But, as Dr. S. has again asserted the infrequency of consumption in cold climates, as if his arguments on this point had not been answered, it is necessary for me to say, that this conclusion is founded entirely on evidence drawn from the writers on one side of the question, whilst the evidence on the other side Dr. S. has entirely neglected, as may be seen by what I have stated above, as well as by the observations in my former letter.

Respecting the other two positions of Dr. Sutton, I think it hardly worth while to say any thing; yet, every medical man is perfectly aware, that in most instances where a disorder arises from any peculiarity of place, the same causes which produced it, will generally tend to promote its progress and to hinder its cure. Thus agues are produced in marshy districts of our own country, and a continuance in those districts will, sometimes, render every mode of treatment abortive, so that the patient to be cured, must be removed to another place. Remittents, dysenteries, liver complaints, the diseases of hot countries, are often relieved, or even cured by a removal to a colder climate; and, on the same principle, I believe, that consumption, during the period when it admits of cure, may frequently be very successfully opposed by exchanging the cold and variable weather, by which, in so many instances, it is produced and supported in this country, for a warmer and more equable temperature.

The

The remaining arguments of Dr. Sutton are the following:—I. That consumption is divided into two stages. II. That, in the latter stage, the disease proceeds more quickly to its termination in the West Indies and in the Mediterranean, than in colder climates. III. That patients will apply at the infirmary for diseases of the lungs only during the latter stage; and, consequently, the warmth of the infirmary will be hurtful rather than beneficial to them.

I. I have no objection to Dr. Sutton's division of consumption into two stages; but my readers very well know, that all such divisions are arbitrary, and that the boundaries between these stages may be differently fixed by different individuals. I should, therefore, have been glad if Dr. Sutton would have defined his terms before he proceeded to argue on them.

II. To Dr. Sutton's second argument, I have not the slightest hesitation in granting, that the latter stage of consumption proceeds more quickly to its termination in the West Indies, and in the summer of the Mediterranean, than in a colder temperature. This circumstance I conceded in my former letter, and further stated that I had occasionally noticed the same to occur in this country, when the weather in summer continued during several days to be much hotter than usual.

But this argument of Dr. Sutton's, and this concession on my part can avail him very little, because

1. They apply merely to the latter stage of the disease, and not to the former stage. If the various testimonies, which Dr. S. has brought forward on this point, be examined, it will be found that all, excepting Dr. Burnett, speak of the latter period of consumption; and, I shall presently have occasion to remark, that his solitary testimony respecting the earlier periods of the disorder is abundantly outweighed by the testimony of other medical observers; indeed, Dr. S. acknowledges that it is chiefly against this stage of the complaint that his arguments are directed. But every practitioner knows full well, that our modes of treating disorders vary at their different periods, and that a very high temperature hastening the progress of consumption in the latter stage, will by no means prove it to be prejudicial before that stage arrives.

2. When consumption has arrived at its latter stage, it is very rarely cured, be the temperature or treatment what they may. Every practitioner occasionally meets with an instance, in which consumption has apparently advanced very far, and yet the patient recovers. But this is so rare, that it is merely considered as an exception to a general rule, and not the result of any peculiar mode of treatment. What, therefore, is beneficial in the early stage of the disease, when probably there may be a chance of recovery, is of of much more consequence than the palliation of symptoms, which is usually all that can be done in the latter stage. If Dr. Sutton can cure consumption when advanced to this period, I will gladly acknowledge his great superiority to myself and to all my professional brethren, and will most thankfully receive his instructions.

3. I do not recommend a *hot* but a *warm* equable temperature in consumption. Dr. Sutton must know, that the temperature which I have recommended in consumption is that of moderate warmth, that of about 65°. This I have repeated so frequently, that it is utterly impossible Dr. S. could have overlooked it. But this is not the temperature of the summer in the Mediterranean, nor of the West Indies at any period. There is a wide differences between 65° and 75° or 80°. Under the former, we generally in this country feel comfortably warm without exercise, but do not feel hot; most persons wish to approach a fire if the thermometer stands at 60°; at 75°, most individuals will perspire very freely even when sitting still. The temperature of 65° is a moderate summer temperature of our island. Dr. S. has not attempted to shew, that in the moderate summer-weather of England patients sink rapidly during the latter stage of the disorder; yet he, as well as every other practitioner, must have seen many cases of consumption in all its stages, both in summer and in winter. Some of these he would have been glad to quote, if he had found them at all to his purpose, instead of travelling to the Mediterranean and to the West Indies to cite instances, which instances, after all, are not to the point, as being placed in a temperature above that which I recommend. Dr. Sutton supposes, that consumption continues about six months. It is allowed by all parties, even by Dr. S. that the greatest number of cases have their origin in winter; they ought then to arrive at their close during the warm weather of summer; yet, we find, by the registers, that little more than half as many persons die of consumption in July and August as in January and February. The temperature, therefore, of 75° or 80° hastening the death of patients in the latter stage of consumption will prove nothing against the temperature of 65° in the same disorder, and in the same stage of the disorder. Hence, Dr. Sutton's argument demonstrates nothing against my position, that a moderately warm temperature is generally beneficial in consumption.

4. Warmth of temperature is beneficial in the former stage of consumption, during which the disease occasionally is curable; in this stage even heat appears, in many instances, to be useful, at least not to be hurtful. That

 warmth warmth is beneficial is shewn by the very authors quoted by Dr. Sutton. Dr. Gourlay, speaking of Madeira, says, (p. 90, &c.) that it is "the favourite retreat of consumptive patients from the northern parts of Europe. Here the unhappy sufferers under this formidable disease cheat the winter of their own climate, and gain that cessation of suffering which such a situation is fitted to produce;" and, again, that Madeira is "highly beneficial in this disease with the natives of this country." He then laments that in general a removal is too long delayed. Why does he lament, but because a removal at an early period is beneficial, at a later period not so? He says, (p. 96) "This unusual success, (which he occasionally met with in consumption) I ascribe, in a great measure, to the concomitant advantages in the benignity of the climate, and also to a constant attention on my part to palliate the uneasy symptoms." Lempriere, as quoted by Dr. Sutton, p. 91, Medical and Physical Journal for August 1815, says, "Patients, when in the incipient state, or rather when predisposed to the disorder (consumption) in this country, may with reasonable hopes be sent to the West Indies." Irvine, speaking of Sicily, (p. 93) says, "I have seen the progress of many cases of phthisis arrested, and many men recover, for a time at least, a perfect state of health, who would in all probability have died in England." A plain man, unwarped by preconceived opinions, would suppose that this passage spoke favourably of the Sicilian climate, as preferable in consumption to the climate of England. But Dr. Sutton has endeavoured to twist this into a testimony in favour of the English over the Sicilian climate. He says, "I leave it to every one to draw his own conclusions, why these men recovered only for a time at least. In England, although the disease is considered very fatal, we see patients recover from an apparent state of confirmed consumption to a perfect re-establishment of health!" Dr. Irvine, in another passage, confirms what he had before said of the superiority of the Sicilian climate, (p. 99,)—"The phthisical invalid will find the Sicilian climate, if not the best in the world, at least greatly more congenial to his frame than the fogs and rains of England."

To counterbalance these testimonies, Dr. Sutton produces the evidence of Dr. Burnett, whom he states as writing—"With respect to consumptive complaints receiving benefit from a residence in the Mediterranean, I can only say, that, out of many hundreds, I never saw one, during any season of the year, whose case was in any shape ameliorated by it." This is very strong language indeed, and appears to me to imply, that every consumptive case must, in the Mediterranean, ranean, proceed to a fatal termination; for it includes every period of the complaint, from its commencement to its close. I candidly confess, that, if this passage were not so very strong, and so very general, I should be inclined to pay a much greater degree of attention to it. However, I leave it to the reader to determine whether he will credit a solitary witness on one side, or three equally credible witnesses on the other side of this question. Although Dr. Sutton has asserted that warmth of temperature, "as a general rule, is disadvantageous" in consumption, the testimony of Dr. Burnett is the only argument in his letter by which it is attempted to be proved hurtful in the former stage of the complaint.

I had purposed citing several cases where I have known a change of climate attended with the greatest benefit in consumption; but want of room compels me to omit them. I can, however, assert the fact, that in several instances such a change has been of the greatest possible advantage.

That a warm temperature of the atmosphere is in this country generally beneficial in the early stage of consumption, I can state from a considerable number of observations. Dr. Cullen, in his "First Lines," No. 896, says, "In this climate they (the symptoms of consumption) very often take up some years, the symptoms appearing especially in the winter and spring; commonly becoming easier, and sometimes disappearing, during the summer, but returning again in winter: they at length, after two or three years, prove fatal towards the end of spring, or beginning of summer." My own experience, and I believe that of my readers, perfectly agrees with this remark of Cullen. I have known many such invalids, who, could winter have been banished from our island, would almost certainly have escaped the advance of the complaint. Also, when the disease had commenced, and had made such progress as to be clearly recognised, I have frequently observed patients to vary with the thermometer. In severe weather, the cough, and every other symptom of the complaint, has been aggravated. When the temperature of the atmosphere has become milder, the symptoms have likewise been mitigated. In many cases, this has been so decided during winter, that the patient's fate has appeared to me to depend on the future mildness or severity of the weather.

In this country, consumption is much more fatal in winter than it is in summer. This argument I before produced, and, in my letter in your Journal, showed that nearly double the number of persons died in January and February of those who died in July and August. This I conceived to arise arise from the difference of temperature between summer and winter. Dr. Sutton could not contradict the fact; but asserts it to depend on the patients breathing "an atmosphere much charged by fire, and by frequent respiration." Such is Dr. Sutton's assertion; but he has given no argument to prove his assertion. Dr. Sutton does not venture to affirm that the disease is produced by persons breathing such an atmosphere; but, I believe, allows that the cold and variable weather of winter occasions the commencement of consumption to be more frequent in that season than in summer. It is only, then, after the disease is formed, that he considers the changed atmosphere to be thus prejudicial. As Dr. S. has not attempted to prove his assertion, I am merely to consider it as an opinion totally unestablished. My reasons for maintaining that the greater number of deaths in winter depends on the difference of temperature, are—1. Because consumption prevails more in this country than in warmer latitudes. 2. Because it commences much more frequently in winter than in summer. 3. Because, after the disease is formed, its symptoms during winter are generally aggravated in cold, and diminished in mild, weather. 4. Because I have, in several instances, known the disorder stopped in its progress by the patient removing to a mild climate. 5. Because I have frequently known artificial warmth of temperature beneficial in consumption. 6. Because, during part of the disorder, it is frequently difficult to distinguish it from asthma or protracted catarrh, which is almost invariably benefitted by warm temperature.

III. Dr. Sutton further affirms, that patients will apply at the Infirmary for diseases of the lungs only during the latter stage of consumption; that heat, being prejudicial in that stage, the Infirmary, instead of promoting their recovery, will hasten their dissolution. I have just shewn that, though *heat* may be prejudicial in the latter stage of the disorder, Dr. S. has been unable to shew that moderate *warmth* is prejudicial in any period of consumption. Indeed I think I have proved that, as a general rule, warmth of temperature is beneficial in this disease. As there are exceptions to all general rules, so there are exceptions here; but, as a general rule, I believe it to be correct. Dr. S. affirms that patients will apply at the Infirmary only during the latter stage of the complaint; but gives no proof of his assertion; and then argues, on that unproved position, that the Infirmary cannot be useful. Against Dr. S.'s assertion I can bring fact, and can say that persons in both the stages of the disorder apply for admission. The worst cases have been uniformly received, unless they evidently appeared to be sinking under the the effects of the disease; and, where their sinking has allowed of a doubt, they were invariably admitted. Yet, in several instances, I have had the greatest reason to think, that the patients experienced a degree of amelioration which they could not possibly have enjoyed had they not possessed the advantage of a warm temperature. I will relate a few cases, in which warm temperature was employed, and which will serve to illustrate what I have just asserted.

Dr. Trotter, in Medicina Nautica, vol. iii. p. 346, note, says, "Dr. Cullen used to relate the case of a gentleman of Philadelphia, in phthisis, who passed his winters in the West Indies, by way of relief; but, happening one season to miss his passage, he fell upon the expedient of keeping within doors, in a room heated to the temperature of Jamaica, and found similar relief, till the summer returned."

In the Monthly Magazine for March of this year, p. 121, I have related several cases which were treated during winter by a warm temperature. These were abridged from cases given in a former Number of the Medical and Physical Journal. John Osborn, No. I., I considered as a case of incipient consumption, which was relieved by the use of a warm temperature. I saw this young man the August after he had been under my care. He then was strong and hearty, and as capable of working as ever he had been in his life. But the ensuing winter I again attended him, but now as a dispensary patient. At this time he was not so ill as when he first applied to me the year preceding. The treatment was on exactly the same plan as that of the former winter, excepting that he now had not the advantage of the warmer apartment. The consequence of this difference was, that he died. I am convinced, from what I witnessed the winter before, that, could he have breathed an atmosphere at the temperature of 65°, he would again have recovered.

Benjamin Palmer, mail-coach guard, aged 27, ill six weeks, was admitted into the Infirmary Nov. 19, 1814. He considers his disorder to have arisen from his having been drenched with rain whilst guarding the mail, and from want of subsequent care. Coughs and expectorates considerably, much of which is mucus, but a portion of it decidedly pus; the pus of greenish yellow colour; breath short; appetite not good; some pain in the throat and in the chest; very hoarse; much emaciated; extremely weak; p. 120, and weak. This man gradually amended, and was discharged Jan. 11, 1815, having been in the house seven weeks. At that time he had scarcely any cough; expectoration trifling; no pus in it; his flesh much increased; strength and appetite good. After quitting the Infirmary, he was constantly exposed to the open air in looking out for a situation. Although the weather was severe during a part of this time, yet at the end of three weeks he called on me to inform me that he was still very well, and was just about to go into the country.

William Slade, tailor, aged nineteen, ill five weeks, thin and narrow-chested, admitted March 1, 1815. Cough not very severe; expectoration neither purulent, nor large in quantity, but thrown up with difficulty; pain in left shoulder and left side of sternum when he coughs; breath very short; occasionally is chilly, occasionally hot; perspires considerably, chiefly during sleep; appetite bad; much emaciated; very weak; nose bleeds occasionally; is becoming worse; has not worked during ten days. Discharged March 22d, having been in the Infirmary three weeks. When discharged, he had not a vestige of the disease in his chest remaining; his strength, appetite, and flesh, much increased.

Richard Martin, aged 16, ill twelve years, admitted March 22d. Small of his age; his chest narrow and deformed, the sides of this part being concave rather than convex; coughs much, chiefly in the morning; expectorates nearly a pint daily, expectoration completely purulent; spat some blood a month ago, and two years before had done the same; pain in his chest; breath very short; perspires greatly at night; excessively weak; very much emaciated; always feels hot; thirsty; constantly worst in cold and foggy weather. Discharged April 19th, four weeks after his admission, in consequence of the Infirmary being then shut for in-patients. Expectoration still purulent; about one-sixth of what it had been on his admission; appetite good; countenance much improved in appearance; emaciation not nearly so great; strength considerably increased; very little cough; no pain in the chest.

The cases just related as having been in the Infirmary were seen not merely by myself, but by Mr. Shillitoe, the apothecary of the Infirmary, by the members of the committee (some of whom were medical practitioners), and by many persons who visited the establishment. I much regret that Martin did not come into the Infirmary a month before the time when he was admitted, or that we could not retain him longer, as the benefit which he received was so extremely marked. If Dr. Sutton's unproved assertion were fact, that "Dr. Buxton's notions of the treatment of this disease" are so very prejudicial, these patients ought to have been destroyed by being kept in a warm apartment. In fact, I am fully persuaded that Dr. Sutton never attended a patient in the former stage of consumption, whose chamber was preserved during one month at about the temperature of 65°; nor do

I believe

I believe that Dr. S. will assert that he has seen a case of this description. Much less do I believe, if he had witnessed the cases which I have, that he could retain the opinions which from theory he has formed.

The points which Dr. Sutton has chiefly laboured to prove are, that consumption is rare in countries much colder than England, and that the latter stage of consumption proceeds with greater rapidity in hot than in cold climates. The former of these positions, I have shown that Dr. S. has completely failed to establish. The latter is correct; but it proves nothing against my opinions, as I recommend a moderately warm and not a hot temperature.

The positions which I laid down at the commencement of my letters were,—that consumption is rare in hot countries; that it is not unfrequent in mild climates; that it is very frequent in this country; that in this country it is more frequent and fatal in winter than in summer; that it has often been cured or relieved by the use of a warm temperature during winter.

In my letter in the Monthly Magazine, I am aware that I had not fully proved the second of these positions, because the writers on the diseases of mild climates had not given us tables of diseases and deaths in the countries of which they treated, but had spoken of the diseases merely in general terms. I had, however, rendered the position so probable, that I believe most persons who read the letters in the Monthly Magazine, or consult for themselves the author to whom I referred, will draw the same conclusion which I have done, viz. that the number of deaths by consumption in the mild climates holds a middle station between the deaths by this disease in hot countries and in our own.

Dr. Sutton has not produced a single argument that can disprove any one of my positions. He has been enabled chiefly to attack the second position for the reason I have just given. But I think I have shewn that his mode of judging is too partial to enable him to draw a general conclusion. And the positive evidence which I have brought respecting our troops in Spain, Portugal, Gibraltar, and Sicily, is sufficient fully to prove that the deaths by consumption in mild climates are, in proportion, not nearly so numerous as in England.

I have, therefore, a right to consider my own positions as established, whilst the assertions and reasonings of Dr. Sutton do not at all diminish their firmness and stability, as applied to consumption.

With regard to asthma, or chronic catarrh, Dr. Sutton still continues silent, although I had called on him to deliver his opinions opinions respecting this disease, and although the Infirmary for Diseases of the Lungs was intended for asthma as well as for consumption. This silence I have a right to consider as a tacit acknowledgment, on the part of Dr. S., that my positions respecting asthma are correct, and that warmth of temperature during winter is beneficial in this disorder.

*New Broad-street;*
*Sept.* 29, 1815.

P. S.—In consequence of the length to which this letter has been protracted, I have not noticed several passages of minor importance in Dr. Sutton's communication. I will, however, just mention, that the temperature varies as much in *frequency* and in extent during summer as during winter. I will also observe, that a misrepresentation of an opinion of mine is given by Dr. Sutton, p. 96, which merely requires for its refutation, that the reader should turn to my letter in your Journal for May, p. 369, and look at the passage misrepresented.

---

*For the London Medical and Physical Journal.*

